# The predictive value of attenuated proteinuria at 1 year after steroid therapy for renal survival in patients with IgA nephropathy

**DOI:** 10.1007/s10157-012-0744-x

**Published:** 2012-12-06

**Authors:** Keita Hirano, Tetsuya Kawamura, Nobuo Tsuboi, Hideo Okonogi, Yoichi Miyazaki, Masato Ikeda, Masato Matsushima, Kazushige Hanaoka, Makoto Ogura, Yasunori Utsunomiya, Tatsuo Hosoya

**Affiliations:** 1 Division of Kidney and Hypertension, Department of Internal Medicine, Jikei University School of Medicine, 3-25-8 Nishi-Shinbashi, Minato-Ku, Tokyo, 105-8461 Japan; 2 Division of Clinical Epidemiology, Research Center for Medical Science, Jikei University School of Medicine, 3-25-8 Nishi-Shinbashi, Minato-Ku, Tokyo, 105-8461 Japan

**Keywords:** Corticosteroid therapy, Proteinuria, Threshold, Clinical remission, Endocapillary hypercellularity, Tonsillectomy

## Abstract

**Background:**

The relationship between the urinary protein excretion (UPE) initially achieved after steroid therapy and the long-term renal outcome of IgA nephropathy (IgAN) has not been clarified. We investigated the threshold UPE at 1 year after steroid therapy which predicts a favorable renal survival.

**Methods:**

We enrolled 141 IgAN patients who received 6 months of steroid therapy. The endpoint was defined as a 50 % increase in serum creatinine from baseline. The spline model was used to define the threshold UPE predicting renal survival.

**Results:**

Thirteen patients (9.2 %) reached the endpoint at a median follow-up of 3.8 years. When evaluating the relative hazard ratio (HR) of the UPE at 1 year for the endpoint, we found an inflection point at 0.40 g/day on the spline curve. The multivariate Cox model revealed that, in addition to the *Disappeared* category of UPE (range <0.30 g/day), the *Mild* category (range 0.30–0.39 g/day) was associated with more reduced risk of the endpoint [HR 0.02, 95 % confidence intervals (CI) 0.00–0.29] relative to the *Severe* category (range ≥1.00 g/day), whereas the *Moderate* category (range 0.40–0.99 g/day) was not. The estimated glomerular filtration rate <60 ml/min/1.73 m^2^ was also an independent predictor of the endpoint. When renal survival was adjusted with pathological parameters in the Cox model, UPE <0.40 g/day was still an independent favorable predictor (HR 0.08, 95 % CI 0.01–0.45).

**Conclusions:**

In IgAN patients receiving 6 months of steroid therapy, the achievement of proteinuria <0.4 g/day at 1 year could be a therapeutic indicator for a favorable renal outcome.

**Electronic supplementary material:**

The online version of this article (doi:10.1007/s10157-012-0744-x) contains supplementary material, which is available to authorized users.

## Introduction

IgA nephropathy (IgAN), a major component of chronic glomerulonephritis, causes end-stage renal disease in up to 50 % of affected patients [1]. Although proteinuria has been considered one of the most important predictors of renal outcome [2–6], few studies have clarified what degree of proteinuria at an early phase after initial treatment predicts renal survival. Donadio et al. [7] showed a lower amount of proteinuria at 1 year after the introduction of treatment to be associated with a better renal survival. However, they did not define the proteinuria level predicting a favorable renal outcome.

Among the many clinical trials demonstrating the efficacy of steroid therapy for IgAN [8–10], a randomized controlled trial by Pozzi et al. [11, 12] clearly demonstrated that 6 months of steroid therapy significantly reduced the risk of a 100 % increase in serum creatinine from the baseline compared to conventional therapy during a 5- or 10-year follow-up. They demonstrated that the steroid therapy induced the lowest level of proteinuria at 1 year of follow-up.

We herein aimed to define the target level of proteinuria at 1 year after initiating steroid therapy to establish a prognostic threshold for a favorable renal survival of IgAN patients.

## Subjects and methods

### Patients and study design

We collected the medical records from 169 patients with IgAN who received 6 months of steroid therapy between 2004 and 2010 in four affiliated hospitals of Jikei University School of Medicine, employing a historical cohort design. Four patients followed for <1 year after the introduction of steroid therapy were excluded. Another 24 patients who were recruited into a prospective randomized controlled trial were also excluded. Finally, the data obtained from 141 patients were analyzed to elucidate the renal outcome. The patients were followed up until April 2012 or the last day of serum creatinine measurement before April 2012. The cohort study was conducted in accordance with the Declaration of Helsinki, and approved by the Medical Ethics Committee of Jikei University School of Medicine.

### Definitions

The endpoint was defined as a 50 % increase in serum creatinine from baseline. Disappeared proteinuria or hematuria was defined as a urinary protein excretion (UPE) <0.3 g/day or having urinary sediment of red blood cells (U-RBC) <5/high power field (hpf). Clinical remission was defined as the disappearance of both proteinuria and hematuria. The estimated glomerular filtration rate (eGFR) was calculated by the Japanese eGFR equation based on age, sex and serum creatinine [13]. Uncontrolled hypertension was defined as arterial blood pressure (BP) ≥130/80 mmHg [14]. Smoking status was defined according to a report by Yamamoto et al. [15].

### Treatment

The 6-month steroid therapy was previously reported by Pozzi et al. [11, 12], and was modified for Japanese patients as follows: the patients received 0.5 g of methylprednisolone intravenously for three consecutive days at the beginning of the steroid course and again 2 and 4 months later; they were also given oral prednisolone at a dose of 0.5 mg/kg every other day for 6 months. Some patients received a tonsillectomy for chronic tonsillitis complicated with IgAN just before the 6 months of steroid therapy. The patients were administered angiotensin-converting enzyme inhibitors or angiotensin receptor blockers (RAAS inhibitors) and antiplatelet agents as needed.

### Histology

To examine the impact of pathological changes on renal survival, renal biopsy data were obtained if a biopsy was performed within 1 year before corticosteroid therapy. All renal biopsy specimens were processed routinely for light microscopy. Sections were stained with hematoxylin and eosin and periodic acid–Schiff, together with silver methenamine and Masson’s trichrome. Pathological variables were evaluated according to the Oxford classification [16]. “Histological grade (HG)” recently reported from the Special Study Group on Progressive Glomerular Disease in Japan was also adopted in this study [17]. Briefly, four histological grades, HG 1, HG 2, HG 3 and HG 4, were established corresponding to <25, 25–49, 50–74 and ≥75 % of glomeruli exhibiting cellular or fibrocellular crescents, global sclerosis, segmental sclerosis or fibrous crescents.

### Statistical analyses

Normally distributed variables were expressed as the mean ± standard deviation (SD) and compared using the *t* test or one-way ANOVA. Nonparametric variables were expressed as medians and interquartile ranges (IQRs) and compared using the Mann–Whitney *U* test, Kruskal–Wallis test, Spearman correlation or Friedman test. Categorical variables were expressed in percentages and compared using the chi-squared test.

To identify a threshold UPE at 1 year that predicts a favorable outcome, we first specified the median UPE for each decile. Second, using the highest decile as the referred category, the relative hazard ratios (HRs) adjusted by the baseline eGFR were plotted according to the specified median values of each decile. Third, quadratic splines were fitted to the relative HR with knots. The spline model is considered to be a smooth function that is sensitive to changes in the relationship between a predictor variable and an outcome across the range of the predictor [18]. The UPE was log-transformed for the spline analyses. The result of the threshold analysis was additionally ascertained by a receiver operating curve (ROC) analysis.

Renal survival was analyzed using the Kaplan–Meier method. In addition, it was analyzed in multivariate Cox regression models to explore the independent prognostic value of predictors. The variables with *p* value <0.1 in the univariate analysis were selected as predictors for the multivariate model. The start point of follow-up was 1 year after steroid therapy in Cox–hazard models. Different relevant multivariate models were tested, obeying the standard statistical rules. The results were expressed as HR with 95 % confidence intervals (CI).

Values of *p* < 0.05 were considered to be statistically significant. All statistical analyses were performed with IBM SPSS Statistics ver. 19.0 software (Chicago, IL, USA).

## Results

### Baseline characteristics and outcome

The clinical and pathological characteristics at baseline and the outcomes are presented in Table 1. The median initial proteinuria was 1.00 g/day, and the mean eGFR was 72.8 ml/min/1.73 m^2^. During a median follow-up of 3.8 years (IQR 2.5–5.3), 13 patients (9.2 %) reached the endpoint. One hundred and eighteen patients (83.7 %), who underwent a renal biopsy within 1 year before the steroid therapy, had clinical backgrounds similar to the overall patients.

**Table 1:** Baseline characteristics and outcomes of the 141 patients analyzed in the study

Variables	Overall (*N* = 141)	Patients who received RBx within 1 year before treatment (*N* = 118)
Baseline features		
Age (years)	34 (26–43)	35 (27–43)
Female	72 (51.1)	58 (49.1)
Current smokers	34 (24.1)	27 (22.9)
BP ≥130/80 mmHg	43 (30.5)	40 (33.9)
UPE (g/day)	1.00 (0.65–1.70)	0.94 (0.63–1.67)
U-RBC		
≥30/hpf	77 (54.6)	66 (55.9)
5–29/hpf	58 (41.1)	46 (39.0)
<5/hpf	6 (4.3)	6 (5.1)
eGFR (ml/min/1.73 m^2^)	72.8 ± 28.0	71.6 ± 28.7
eGFR <60 ml/min/1.73 m^2^	51 (36.2)	45 (38.1)
Concurrent treatments		
Tonsillectomy	68 (48.2)	48 (40.7)
RAAS inhibitors	62 (44.0)	52 (44.1)
Oxford classification		
M1	–	38 (32.2)
E1	–	74 (62.7)
S1	–	96 (81.4)
T0/T1/T2	–	93/20/5 (78.8/16.9/4.2)
Ext, present	–	108 (91.5)
HG^a^		
HG1/HG2/HG3 + 4	–	32/56/30 (27.1/47.5/25.4)
Follow-up		
Period (years)	3.8 (2.5–5.3)	3.8 (2.3–5.3)
Outcome	13 (9.2)	10 (8.5)

Values are presented as numbers (%), medians (IQR) or mean ± SD

*RBx* renal biopsy, *BP* blood pressure, *UPE* urinary protein excretion, *U-RBC* urinary sediments of red blood cells, *eGFR* estimated glomerular filtration rate, *RAAS* renin–angiotensin–aldosterone system, *M* mesangial hypercellularity, *E* endocapillary hypercellularity, *S* segmental sclerosis, *T* tubulointerstitial atrophy/fibrosis, *Ext* extracapillary lesion, *HG* histological grade

^a^According to Ref. [17]

### Changes in proteinuria during follow-up, and clinical remission rate at 1 year after steroid therapy

**Fig. 1:**
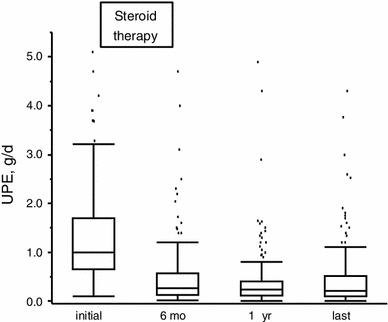
Changes in proteinuria at baseline, 6 months, 1 year and at the last follow-up. The *lines* in the middle and those delimiting the *boxes* indicate the median, 25th and 75th percentile values, respectively. The *whiskers* at the ends of the boxes are lines that show the distance from the end of the box to the largest and smallest observed values that are <1.5 box-length from either end. *Dots* indicate outliers

As shown in Fig. 1, the median values for UPE were significantly decreased at 6 months, 1 year and the last follow-up. The lowest level of UPE was seen at 1 year, with a 78.2 % (IQR 50.0–88.5 %) reduction of the UPE from baseline. At the 1 year follow-up, 49 patients (34.8 %) had reached clinical remission.

### Threshold proteinuria after steroid therapy predicting the renal outcome

**Fig. 2:**
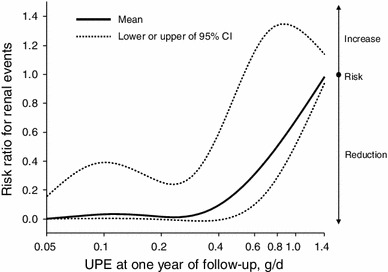
Risk ratio for the endpoint associated with the UPE at the 1-year follow-up. Plots of the risk ratios and 95 % confidence intervals adjusted for the baseline eGFR for the endpoint using the level of proteinuria at the 1-year follow-up examination as the continuous variable are shown (reference: the highest decile, the median of which was 1.44 g/day). The degree of proteinuria was log transformed

We further explored what degree of UPE at 1 year after steroid therapy was associated with renal survival. The spline model of UPE at 1 year was used to predict the relative HR of the endpoint (Fig. 2). The spline curve showed that the relative HRs were equivalent in the range of UPE under 0.4 g/day, but increased as the UPE increased beyond this value, indicating an inflection at approximately 0.40 g/day. Furthermore, the ROC of UPE at 1 year indicated that the optimal cutoff for predicting an unfavorable outcome was 0.40 g/day; the area under the curve and *p* value were 0.78 and <0.001, respectively.

### Categorization of UPE at 1 year after steroid therapy

“Disappeared proteinuria” was previously defined as UPE <0.3 g/day [19] and UPE >1.0 g/day was generally associated with following deterioration of renal function [4–6]. Based on the results from our threshold analysis (0.4 g/day) and the above two values, we divided the UPE at 1 year of follow-up into four categories; *Disappeared* category (<0.30 g/day), *Mild* category (0.30–0.39 g/day), *Moderate* category (0.40–0.99 g/day) and *Severe* category (≥1.00 g/day). The clinical parameters were not significantly different among the four categories, except for the baseline proteinuria (Table 2).

**Table 2:** Baseline characteristics according to the category of proteinuria at 1 year of follow-up

Variables	Category of UPE at 1 year of follow-up (g/day)				*p* value
	*Disappeared* (<0.3)	*Mild* (0.30–0.39)	*Moderate* (0.40–0.99)	*Severe* (≥1.00)	
Number of patients	80	23	22	16	
Age (years)	35 (26–44)	30 (25–42)	32 (26–36)	35 (26–42)	>0.2
Female	39 (48.8)	11 (47.8)	12 (54.5)	9 (56.3)	>0.2
Current smokers	18 (22.5)	5 (21.7)	6 (27.3)	5 (31.3)	>0.2
BP >130/80 mmHg	25 (31.3)	9 (39.1)	5 (22.7)	4 (25.0)	>0.2
UPE (g/day)	0.82 (0.57–1.28)	0.80 (0.64–2.17)	1.58 (0.97–2.28)	1.90 (1.25–2.80)	<0.001^#^
U-RBC >30/hpf	48 (60.0)	12 (52.2)	8 (36.4)	9 (56.3)	>0.2
eGFR (ml/min/1.73 m^2^)	75.1 ± 27.1	73.7 ± 29.1	68.2 ± 29.5	66.3 ± 29.1	>0.2
eGFR <60	25 (31.3)	10 (43.5)	10 (45.5)	6 (37.5)	>0.2
Tonsillectomy	40 (50.0)	10 (43.5)	12 (54.5)	6 (37.5)	>0.2
RAAS inhibitors	35 (43.8)	9 (39.1)	11 (50.0)	7 (43.8)	>0.2

Values are presented as numbers (%), medians (IQR) or mean ± SD

*BP* blood pressure, *UPE* urinary protein excretion, *U-RBC* urinary sediments of red blood cells, *eGFR* estimated glomerular filtration rate. ^#^ *p* < 0.05

### Renal survival according to the UPE category at 1 year by Kaplan–Meier analysis and multivariate Cox model

**Fig. 3:**
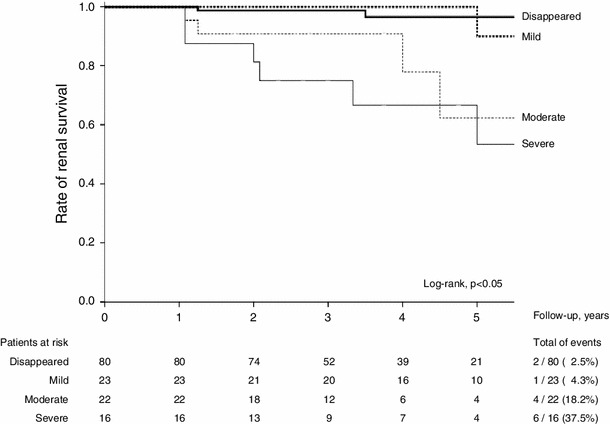
Renal survival determined by the Kaplan–Meier method, stratified by the category of UPE at 1 year after 6 months of steroid therapy. These unadjusted curves demonstrate that, in addition to the *Disappeared* category, the *Mild* category showed significantly better renal survival compared to that in the *Moderate* or *Severe* categories (log-rank, *p* < 0.05 for both strata)

The results of the univariate time-dependent analyses by the Kaplan–Meier method are shown in Fig. 3. Patients in the *Disappeared* and *Mild*categories showed significantly better renal survival compared to the *Moderate* or *Severe* categories (log-rank, *p* < 0.05 for both strata), whereas there was no such difference between the *Moderate* and *Severe* categories (log-rank, *p* > 0.2).

The clinical predictors for the endpoint in the Cox–hazard model are presented in Table 3. Relative to the *Severe* category in the multivariate model, the *Disappeared* and *Mild* categories were favorable predictors, with risk reduction of approximately 90 and 70 %, respectively, whereas the *Moderate* category was not associated with renal survival. In contrast, eGFR <60 ml/min/1.73 m^2^ at baseline was an unfavorable predictor. Clinical remission, as well as a U-RBC <5/hpf at 1 year after steroid therapy, was not associated with renal survival in the univariate model.

**Table 3:** Clinical predictors for a 50 % increase in serum creatinine from the baseline level in the Cox–hazard model

Predictors	Univariate model		Multivariate model^a^	
	HR (95 % CI)	*p* value	HR (95 % CI)	*p* value
At 1 year				
Category of proteinuria^b^				
*Disappeared* ^c^	0.07 (0.01–0.33)	0.001^#^	0.06 (0.01–0.57)	0.014^#^
*Mild* ^c^	0.10 (0.12–0.80)	0.030^#^	0.02 (0.00–0.29)	0.003^#^
*Moderate* ^c^	0.55 (0.16–1.98)	>0.2	0.24 (0.04–1.25)	0.089
U-RBC <5/hpf^d^	2.59 (0.71–9.42)	0.148	–	–
Clinical remission^d^	0.35 (0.08–1.57)	0.170	–	–
At baseline				
Age (years)	1.04 (0.99–1.08)	0.092	1.00 (0.94–1.06)	>0.2
Female^d^	1.06 (0.36–3.16)	>0.2	–	–
Current smoking^d^	3.96 (1.33–11.8)	0.013^#^	1.27 (0.28–5.58)	>0.2
BP ≥130/80 mmHg^d^	1.31 (0.36–4.79)	>0.2	–	–
UPE (g/day)	2.09 (1.43–3.07)	<0.001^#^	–^e^	–^e^
U-RBC ≥30/hpf^d^	0.22 (0.06–0.79)	0.021^#^	0.34 (0.06–1.99)	>0.2
eGFR <60 ml/min/1.73 m^2 d^	11.5 (2.55–52.3)	0.002^#^	24.3 (2.72–217)	0.004^#^
Concurrent treatment				
Tonsillectomy^d^	0.37 (0.11–1.21)	0.099	1.23 (0.27–5.55)	>0.2
RAAS inhibitors^d^	2.06 (0.67–6.29)	>0.2	–	–

*HR* hazard ratio, *CI* confidence interval, *UPE* urinary protein excretion, *U-RBC* urinary sediments of red blood cells, *NE* not enrolled in the multivariate model, *eGFR* estimated glomerular filtration rate, *RAAS* renin–angiotensin–aldosterone system

^a^If the *p* value of the variable was <0.1 in the univariate model, the predictor was selected for the multivariate model

^b^The category is shown in Table 2

^c^Reference = *Severe* category

^d^Yes versus no

^e^As it was related to category of UPE at 1 year (see Table 2), it was not enrolled in the multivariate model

^#^ *p* < 0.05

### Significance of UPE <0.4 g/day as a predictor when the renal survival was adjusted for pathological parameters

The predictive value of UPE <0.4 g/day at 1 year for the outcome when adjusted for pathological parameters in the Oxford classification and “HG” from Japan was examined by the univariate and multivariate models and the data are summarized in Table 4. The univariate analysis revealed that the existence of endocapillary hypercellularity (E1) was significantly associated with a preferable renal survival relative to the absence of endocapillary hypercellularity (E0). T1 or T2 tubular atrophy/interstitial fibrosis was significantly associated with impaired renal survival relative to T0. In addition, HG 2 was significantly associated with favorable renal outcome relative to HG 3 plus HG 4. Although HG 1 was not significantly associated with favorable outcome, no event was observed in 32 patients of HG 1.

**Table 4:** Pathological predictors and UPE <0.4 g/day at 1 year for a 50 % increase in the serum creatinine level from baseline in the Cox model

Predictors	Univariate model		Multivariate model A		Multivariate model B	
	HR (95 % CI)	*p* value	HR (95 % CI)	*p* value	HR (95 % CI)	*p* value
Oxford classification						
M1 versus M0	0.93 (0.24–3.61)	>0.2	–	–	–	–
E1 versus E0	0.23 (0.06–0.89)	0.033^#^	0.44 (0.10–1.91)	>0.2	–	–
S1 versus S0	2.03 (0.26–16.0)	>0.2	–	–	–	–
T1 versus T0	6.97 (1.66–29.2)	0.008^#^	4.35 (1.02–18.5)	0.047^#^	–	–
T2 versus T0	12.8 (2.12–77.1)	0.005^#^	19.1 (2.55–144)	0.004^#^	–	–
Ext, present versus absent	0.44 (0.09–2.06)	>0.2	–	–	–	–
HG						
HG1 versus HG3 + 4	0.00 (0.00–100<)	>0.2	–	–	0.00 (0.00–100<)	>0.2
HG2 versus HG3 + 4	0.24 (0.06–0.92)	0.038^#^	–	–	0.36 (0.08–1.51)	0.161
UPE at 1 year <0.4 g/day^a^	0.10 (0.03–0.36)	<0.001^#^	0.08 (0.01–0.45)	0.004^#^	0.06 (0.01–0.29)	0.001^#^

*HR* hazard ratio, *CI* confidence interval, *M* mesangial hypercellularity, *E* endocapillary hypercellularity, *S* segmental sclerosis, *T* tubulointerstitial atrophy/fibrosis, *Ext* extracapillary lesion, *HG* histological grade, *UPE* urinary protein excretion volume

^#^ *p* < 0.05

^a^Yes versus no

The multivariate model A and model B in Table 4 examined the predictive power of UPE <0.4 g/day at 1 year for renal survival after adjusting for pathological predictors in the Oxford classification and HG, respectively. A UPE <0.4 g/day at 1 year was selected as an independent predictor in both model A and model B.

### Adverse effects

Serious adverse events were not observed during the study period. Although three patients developed type 2 diabetes during the 6 months of treatment, they showed normal levels of glycosylated HbA1 at 1 year with diet therapy alone. Seven patients developed infections during the steroid therapy: five bacterial infections (tonsillitis, pharyngitis) and two viral infections (influenza). Two females became pregnant during the follow-up and maintained a stable renal function.

## Discussion

The goal of this study was to identify the level of proteinuria after steroid therapy associated with a favorable renal outcome in IgAN patients. Previous studies by Reich et al. [4], Hwang et al. [5], or Le et al. [6] have demonstrated that the average level of proteinuria during the whole period of follow-up (A-P) was significantly associated with the renal outcome, providing a targeted proteinuria during long-term follow-up. In contrast, we identified a therapeutic indicator of a favorable renal outcome as an early response to the steroid therapy, which might be more practical than A-P, whereas it was not analyzed in the previous studies. We adopted 1 year as the time to assess the attenuated proteinuria, since another Cox model in our cohort revealed that the values for proteinuria at 1 year were significantly associated with the outcome, whereas those at baseline or 6 months were not (data not shown).

In this study, the spline model revealed that the threshold UPE predicting the outcome was approximately 0.4 g/day. In addition, a multivariate Cox model including the categorized UPE at 1 year revealed that not only the *Disappeared* category but also the *Mild* category were significantly associated with favorable renal survival relative to the *Severe* category. Therefore, attenuated proteinuria <0.4 g/day at 1 year after treatment can lead to a favorable outcome, as well as the disappearance of proteinuria. The predictive power of UPE <0.4 g/day at 1 year for renal survival was confirmed even after adjusting for pathological predictors determined by the multivariate model (Table 4).

Concerning the impact of clinical remission at an early phase on the renal outcome, Tatematsu et al. [20] showed that clinical remission within 2 years after 6 months of steroid therapy was associated with limiting the eGFR decline. In contrast, clinical remission at 1 year was not significantly associated with the endpoint in our univariate Cox model (Table 3). Although the reasons for the discrepancy between the two studies are unknown, there might be several factors responsible. For example, the timing for assessment of clinical remission was different: during the first 2 years in Tatematsu’s study and at 1 year after the intervention in our study. Furthermore, the fact that the incidence of the endpoint in our patients achieving clinical remission at 1 year after the therapy was not significantly different from that in those without clinical remission (4.1 vs. 12.0 %, respectively, *p* > 0.2) may have affected the results shown in Table 3.

Our retrospective study has several limitations. First, we did not include control patients who were followed by supportive therapy alone. Second, the study population and statistical power were small, and the observation period was relatively short to evaluate the outcome in IgAN, leading to the small number of outcomes. Since a limited number of outcomes would generally restrict the number of explanatory variables in multivariate models, we additionally tested the Cox–hazard model for the outcome with two explanatory variables: UPE at 1 year <0.4 g/day and propensity score. The propensity model for UPE at 1 year <0.4 g/day was constructed with the baseline characteristics or pathological parameters. After adjusting the propensity score, we also found the predictive power of UPE at 1 year <0.4 g/day for the outcome (data not shown), suggesting the consistency of the significance of UPE at 1 year <0.4 g/day. Nevertheless, the value of UPE at 1 year <0.4 g/day as a favorable predictor should be ascertained in other studies with longer observation periods and a larger number of outcomes. Third, the role of recurrent proteinuria after 1 year on the progression of IgAN should be examined, since clinical remission was not associated with the endpoint in this study.

In conclusion, the achievement of proteinuria <0.4 g/day at 1 year after 6 months of steroid therapy is an optimal goal for achieving a subsequent favorable renal survival, independent of the baseline renal function or renal pathological changes. Further investigations of the impact of recurrence during follow-up on the endpoint are now in progress.

## Electronic supplementary material

Below is the link to the electronic supplementary material.

Supplementary material (PPTX 112 kb)
