# Supplementary material for: The predictive value of attenuated proteinuria at 1 year after steroid therapy for renal survival in patients with IgA nephropathy
Source: Clin Exp Nephrol. 2012 Dec 6;17(4):555–62. doi: 10.1007/s10157-012-0744-x (PMC3751270; doi:10.1007/s10157-012-0744-x)
Supplement: Supplementary file 1 — Supplementary material (PPTX 112 kb) [file 10157_2012_744_MOESM1_ESM.pptx]

## Slide 1
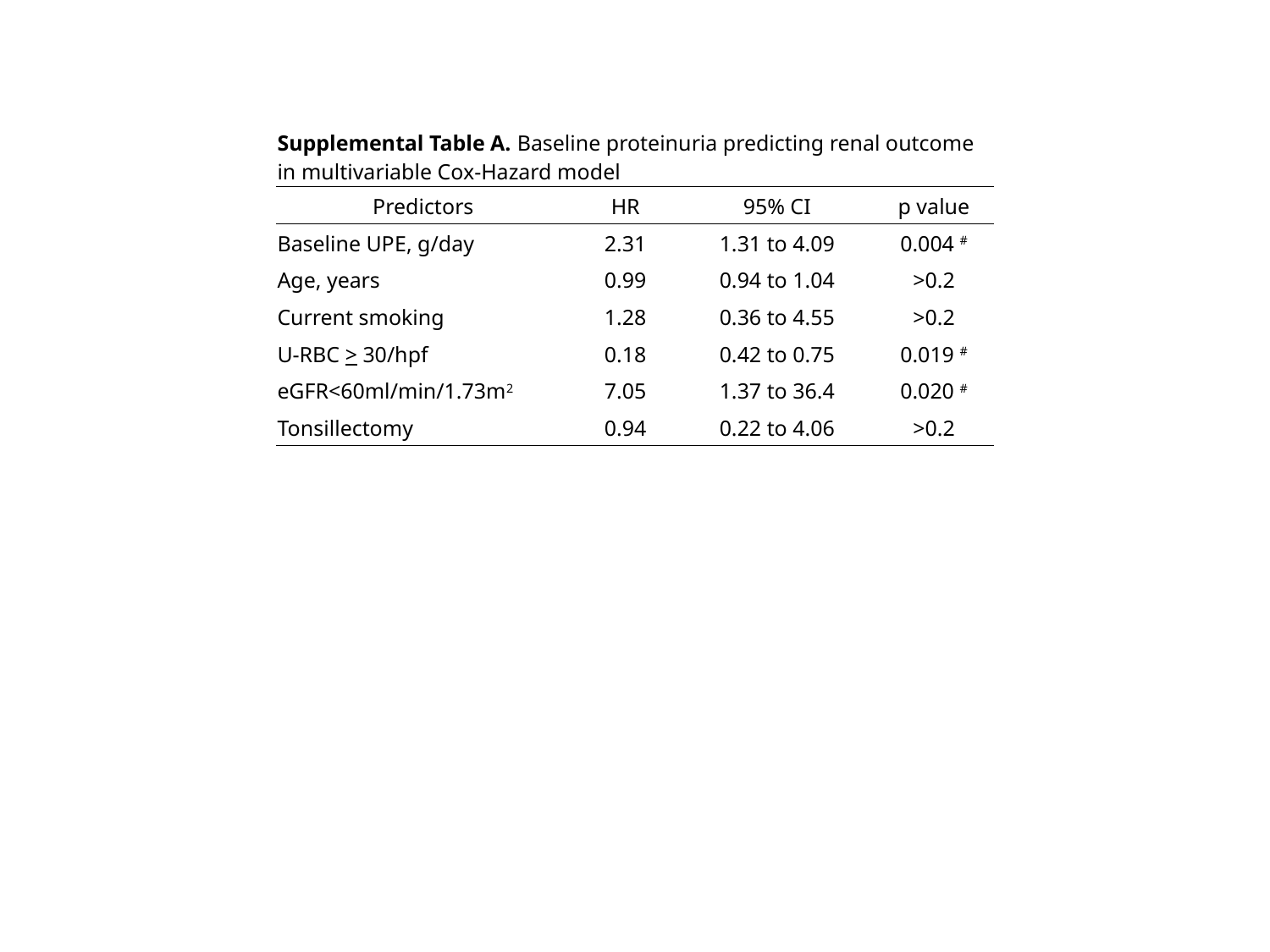

| Supplemental Table A. Baseline proteinuria predicting renal outcome in multivariable Cox-Hazard model | | | |
| --- | --- | --- | --- |
| Predictors | HR | 95% CI | p value |
| Baseline UPE, g/day | 2.31 | 1.31 to 4.09 | 0.004 # |
| Age, years | 0.99 | 0.94 to 1.04 | >0.2 |
| Current smoking | 1.28 | 0.36 to 4.55 | >0.2 |
| U-RBC > 30/hpf | 0.18 | 0.42 to 0.75 | 0.019 # |
| eGFR<60ml/min/1.73m2 | 7.05 | 1.37 to 36.4 | 0.020 # |
| Tonsillectomy | 0.94 | 0.22 to 4.06 | >0.2 |

## Slide 2
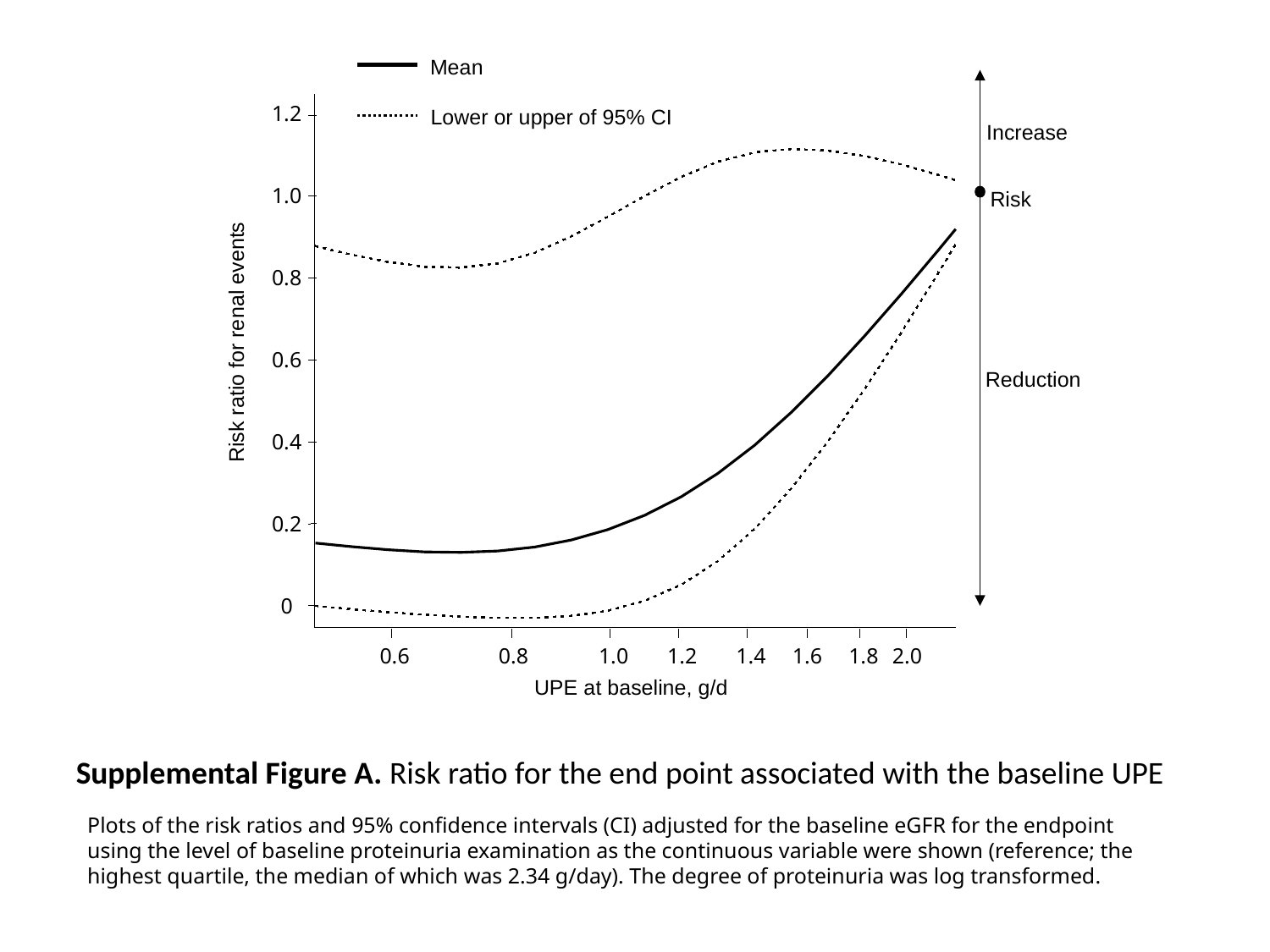

Mean
Lower or upper of 95% CI
1.2
1.0
0.8
0.6
0.4
0.2
0
Increase
Risk
Risk ratio for renal events
Reduction
0.6
0.8
1.0
1.2
1.4
1.6
1.8
2.0
UPE at baseline, g/d
Supplemental Figure A. Risk ratio for the end point associated with the baseline UPE
Plots of the risk ratios and 95% confidence intervals (CI) adjusted for the baseline eGFR for the endpoint using the level of baseline proteinuria examination as the continuous variable were shown (reference; the highest quartile, the median of which was 2.34 g/day). The degree of proteinuria was log transformed.

## Slide 3
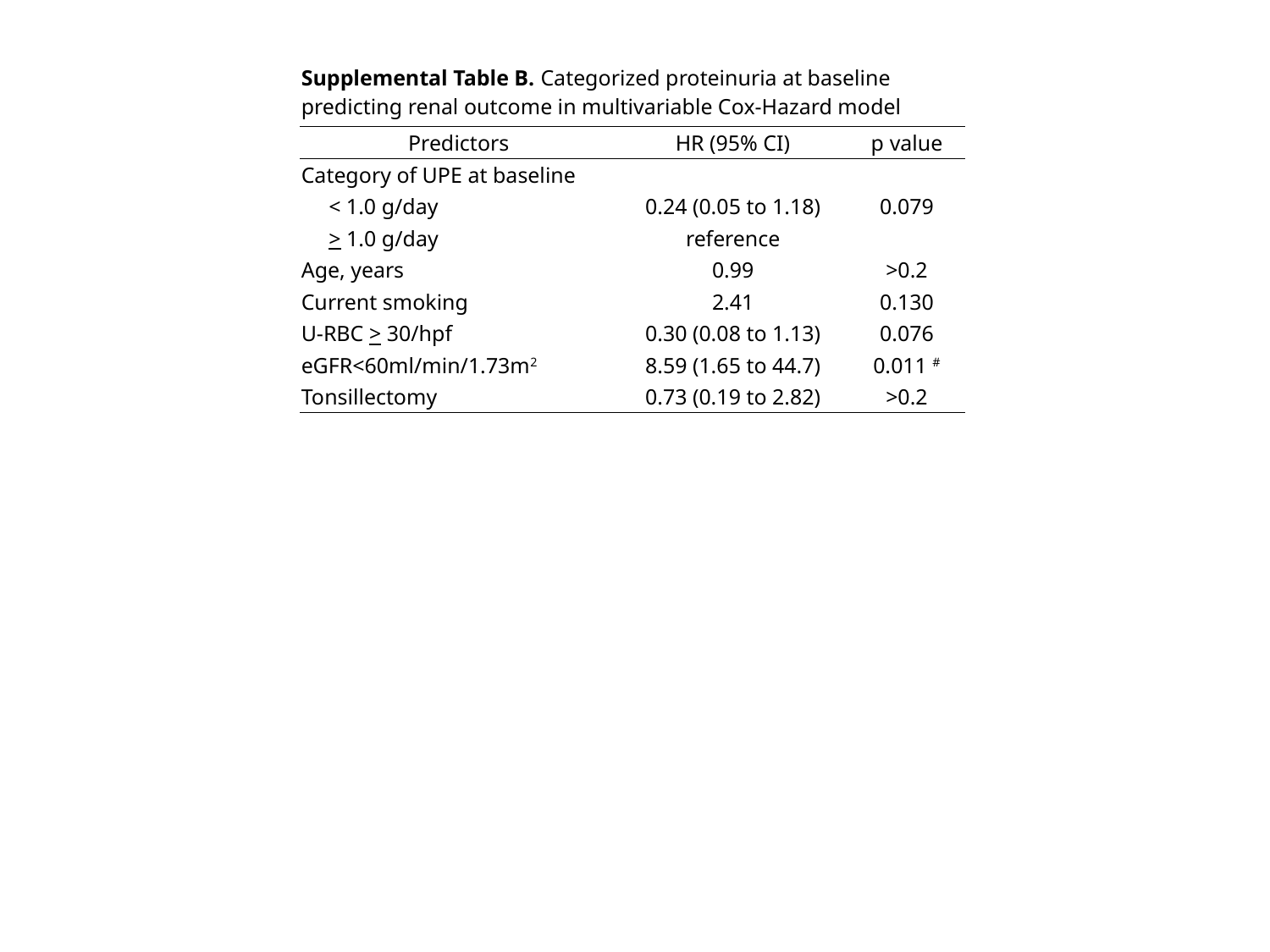

| Supplemental Table B. Categorized proteinuria at baseline predicting renal outcome in multivariable Cox-Hazard model | | |
| --- | --- | --- |
| Predictors | HR (95% CI) | p value |
| Category of UPE at baseline | | |
| < 1.0 g/day | 0.24 (0.05 to 1.18) | 0.079 |
| > 1.0 g/day | reference | |
| Age, years | 0.99 | >0.2 |
| Current smoking | 2.41 | 0.130 |
| U-RBC > 30/hpf | 0.30 (0.08 to 1.13) | 0.076 |
| eGFR<60ml/min/1.73m2 | 8.59 (1.65 to 44.7) | 0.011 # |
| Tonsillectomy | 0.73 (0.19 to 2.82) | >0.2 |

## Slide 4
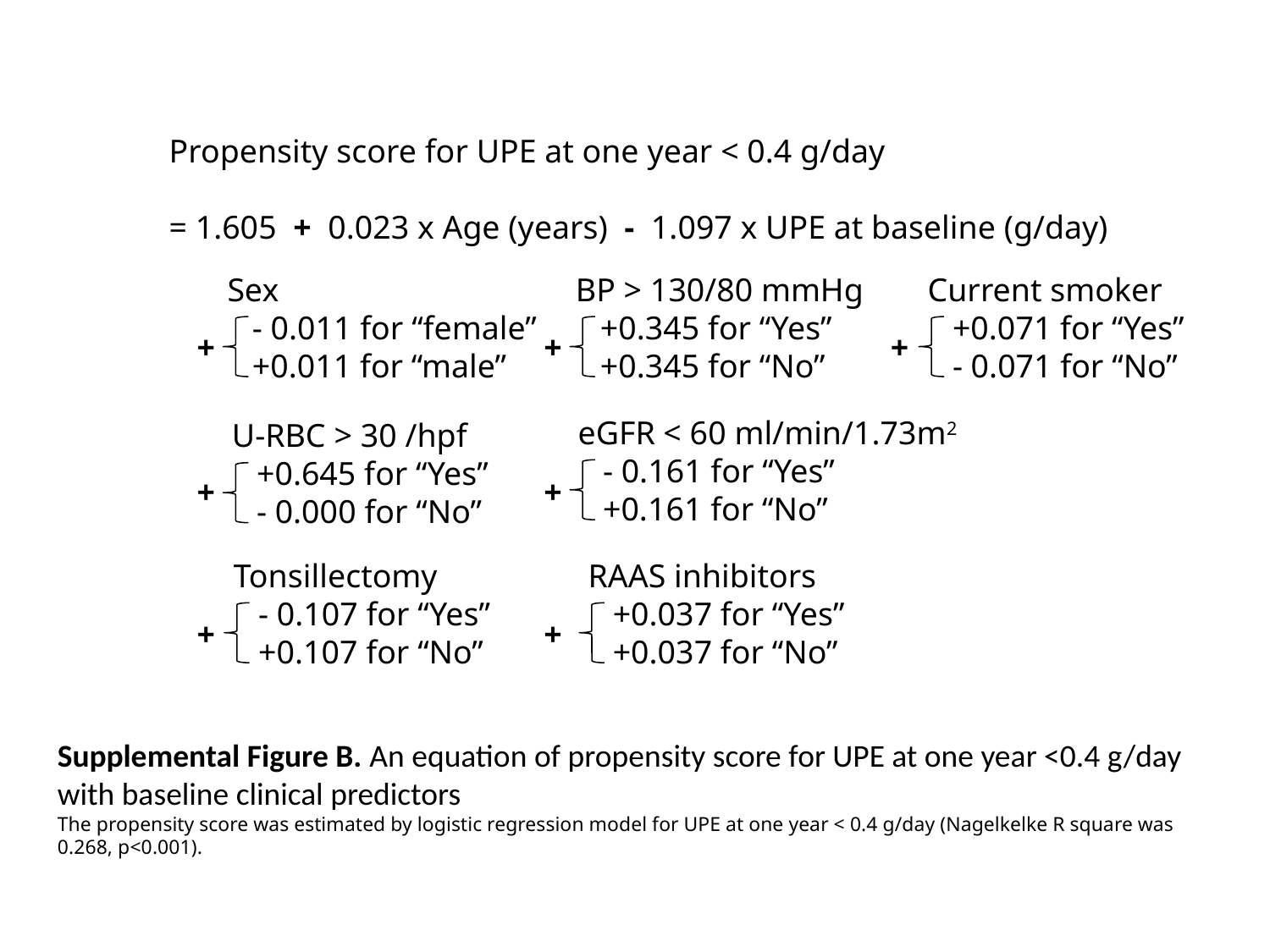

Propensity score for UPE at one year < 0.4 g/day
= 1.605 + 0.023 x Age (years) - 1.097 x UPE at baseline (g/day)
Sex
 - 0.011 for “female”
 +0.011 for “male”
BP > 130/80 mmHg
 +0.345 for “Yes”
 +0.345 for “No”
Current smoker
 +0.071 for “Yes”
 - 0.071 for “No”
+
+
+
eGFR < 60 ml/min/1.73m2
 - 0.161 for “Yes”
 +0.161 for “No”
U-RBC > 30 /hpf
 +0.645 for “Yes”
 - 0.000 for “No”
+
+
Tonsillectomy
 - 0.107 for “Yes”
 +0.107 for “No”
RAAS inhibitors
 +0.037 for “Yes”
 +0.037 for “No”
+
+
Supplemental Figure B. An equation of propensity score for UPE at one year <0.4 g/day with baseline clinical predictors
The propensity score was estimated by logistic regression model for UPE at one year < 0.4 g/day (Nagelkelke R square was 0.268, p<0.001).

## Slide 5
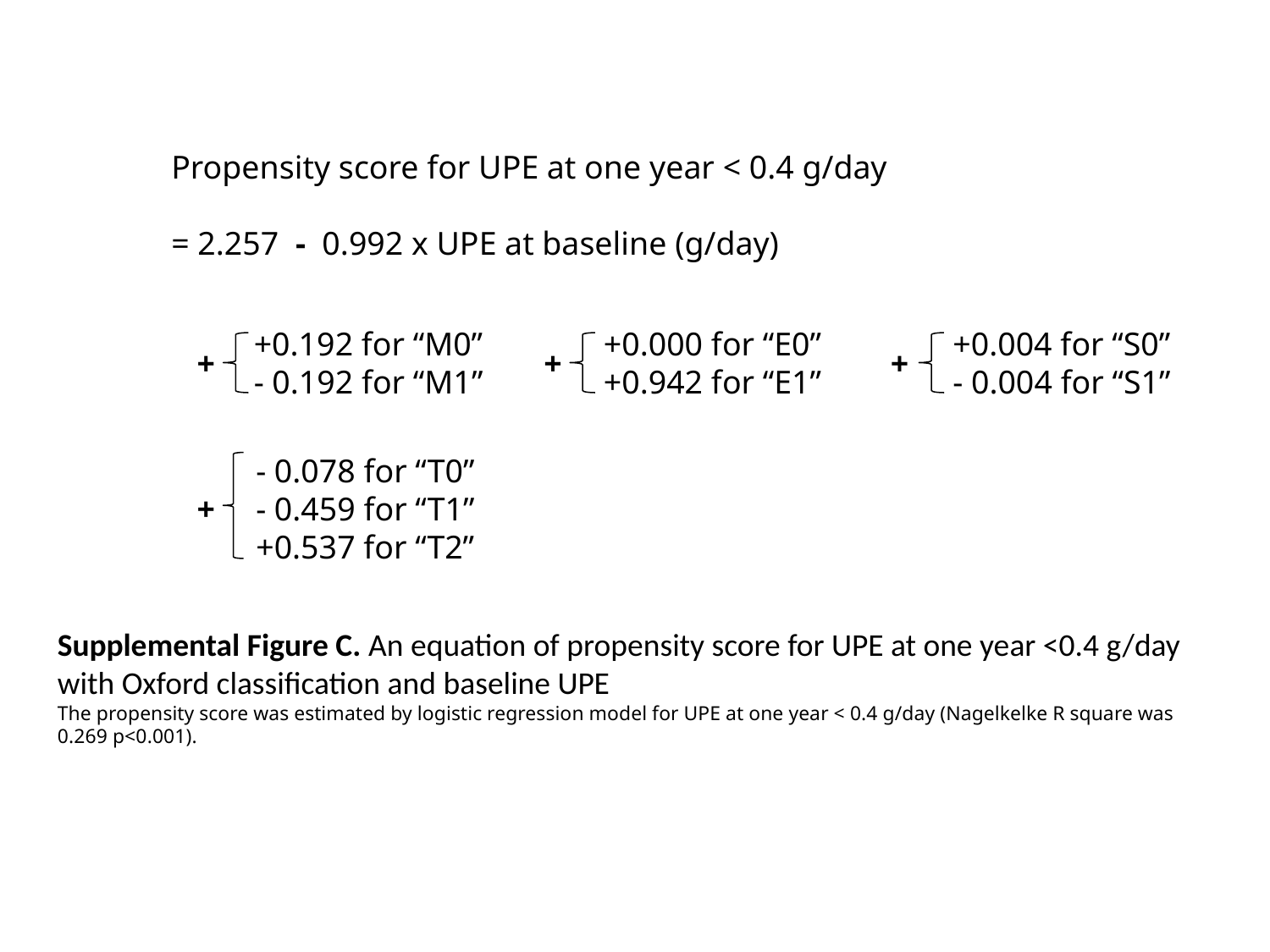

Propensity score for UPE at one year < 0.4 g/day
= 2.257 - 0.992 x UPE at baseline (g/day)
 +0.000 for “E0”
 +0.942 for “E1”
 +0.192 for “M0”
 - 0.192 for “M1”
 +0.004 for “S0”
 - 0.004 for “S1”
+
+
+
 - 0.078 for “T0”
 - 0.459 for “T1”
 +0.537 for “T2”
+
Supplemental Figure C. An equation of propensity score for UPE at one year <0.4 g/day with Oxford classification and baseline UPE
The propensity score was estimated by logistic regression model for UPE at one year < 0.4 g/day (Nagelkelke R square was 0.269 p<0.001).

## Slide 6
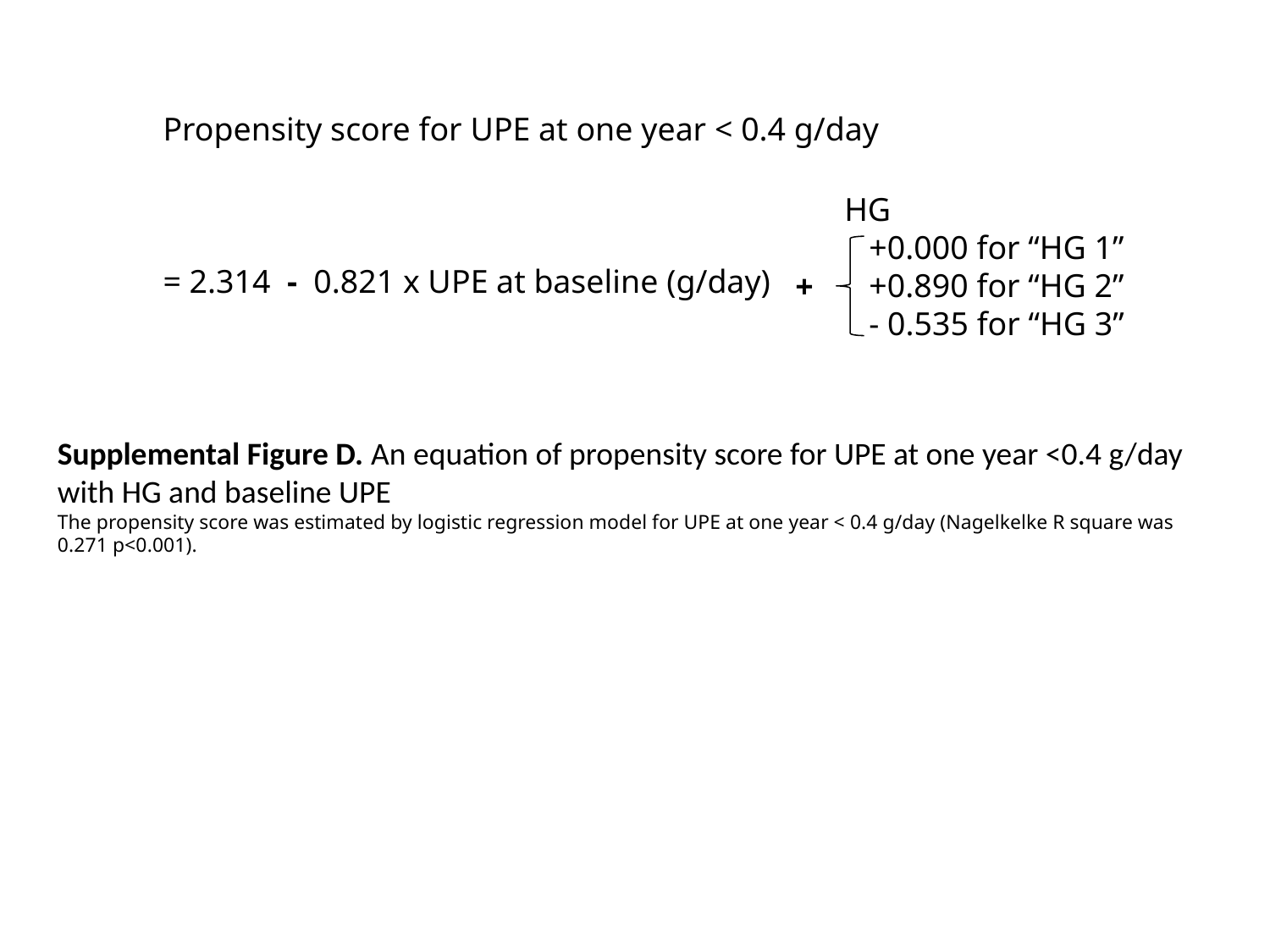

Propensity score for UPE at one year < 0.4 g/day
= 2.314 - 0.821 x UPE at baseline (g/day)
HG
 +0.000 for “HG 1”
 +0.890 for “HG 2”
 - 0.535 for “HG 3”
+
Supplemental Figure D. An equation of propensity score for UPE at one year <0.4 g/day with HG and baseline UPE
The propensity score was estimated by logistic regression model for UPE at one year < 0.4 g/day (Nagelkelke R square was 0.271 p<0.001).

## Slide 7
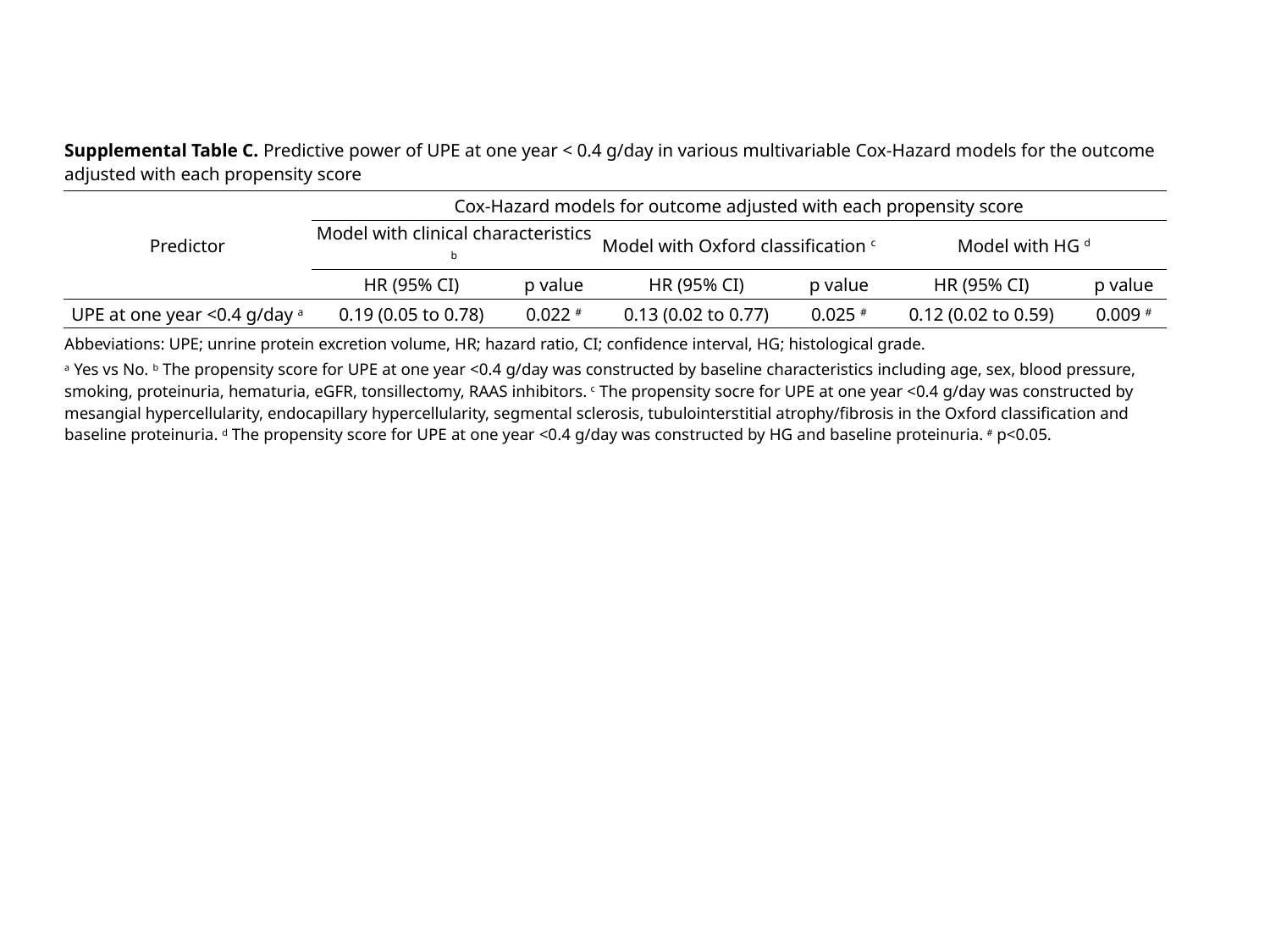

| Supplemental Table C. Predictive power of UPE at one year < 0.4 g/day in various multivariable Cox-Hazard models for the outcome adjusted with each propensity score | | | | | | |
| --- | --- | --- | --- | --- | --- | --- |
| Predictor | Cox-Hazard models for outcome adjusted with each propensity score | | | | | |
| | Model with clinical characteristics b | | Model with Oxford classification c | | Model with HG d | |
| | HR (95% CI) | p value | HR (95% CI) | p value | HR (95% CI) | p value |
| UPE at one year <0.4 g/day a | 0.19 (0.05 to 0.78) | 0.022 # | 0.13 (0.02 to 0.77) | 0.025 # | 0.12 (0.02 to 0.59) | 0.009 # |
| Abbeviations: UPE; unrine protein excretion volume, HR; hazard ratio, CI; confidence interval, HG; histological grade. | | | | | | |
| a Yes vs No. b The propensity score for UPE at one year <0.4 g/day was constructed by baseline characteristics including age, sex, blood pressure, smoking, proteinuria, hematuria, eGFR, tonsillectomy, RAAS inhibitors. c The propensity socre for UPE at one year <0.4 g/day was constructed by mesangial hypercellularity, endocapillary hypercellularity, segmental sclerosis, tubulointerstitial atrophy/fibrosis in the Oxford classification and baseline proteinuria. d The propensity score for UPE at one year <0.4 g/day was constructed by HG and baseline proteinuria. # p<0.05. | | | | | | |

## Slide 8
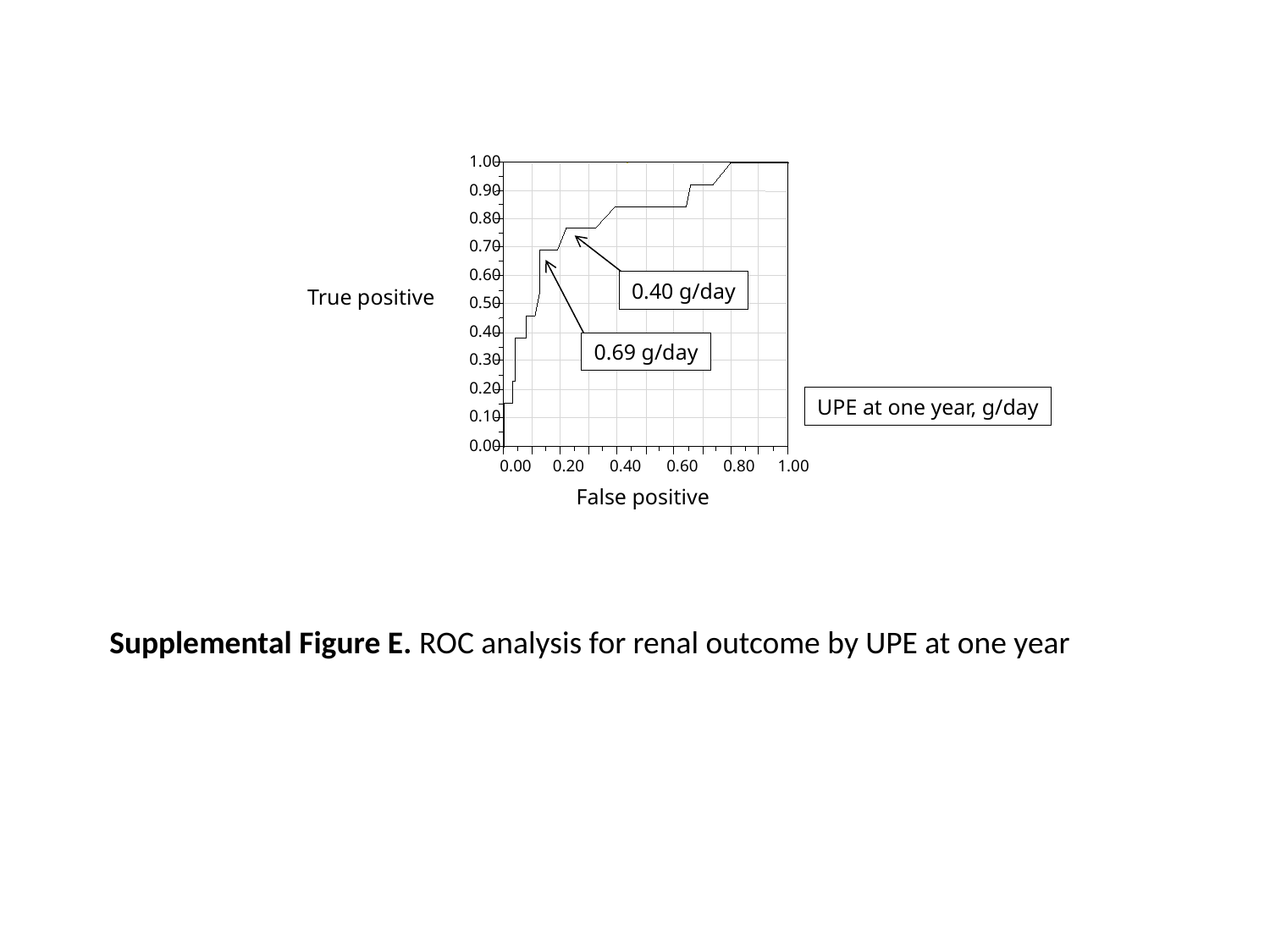

1.00
0.90
0.80
0.70
0.60
0.50
0.40
0.30
0.20
0.10
0.00
0.40 g/day
0.69 g/day
0.00
0.20
0.40
0.60
0.80
1.00
True positive
UPE at one year, g/day
False positive
Supplemental Figure E. ROC analysis for renal outcome by UPE at one year

## Slide 9
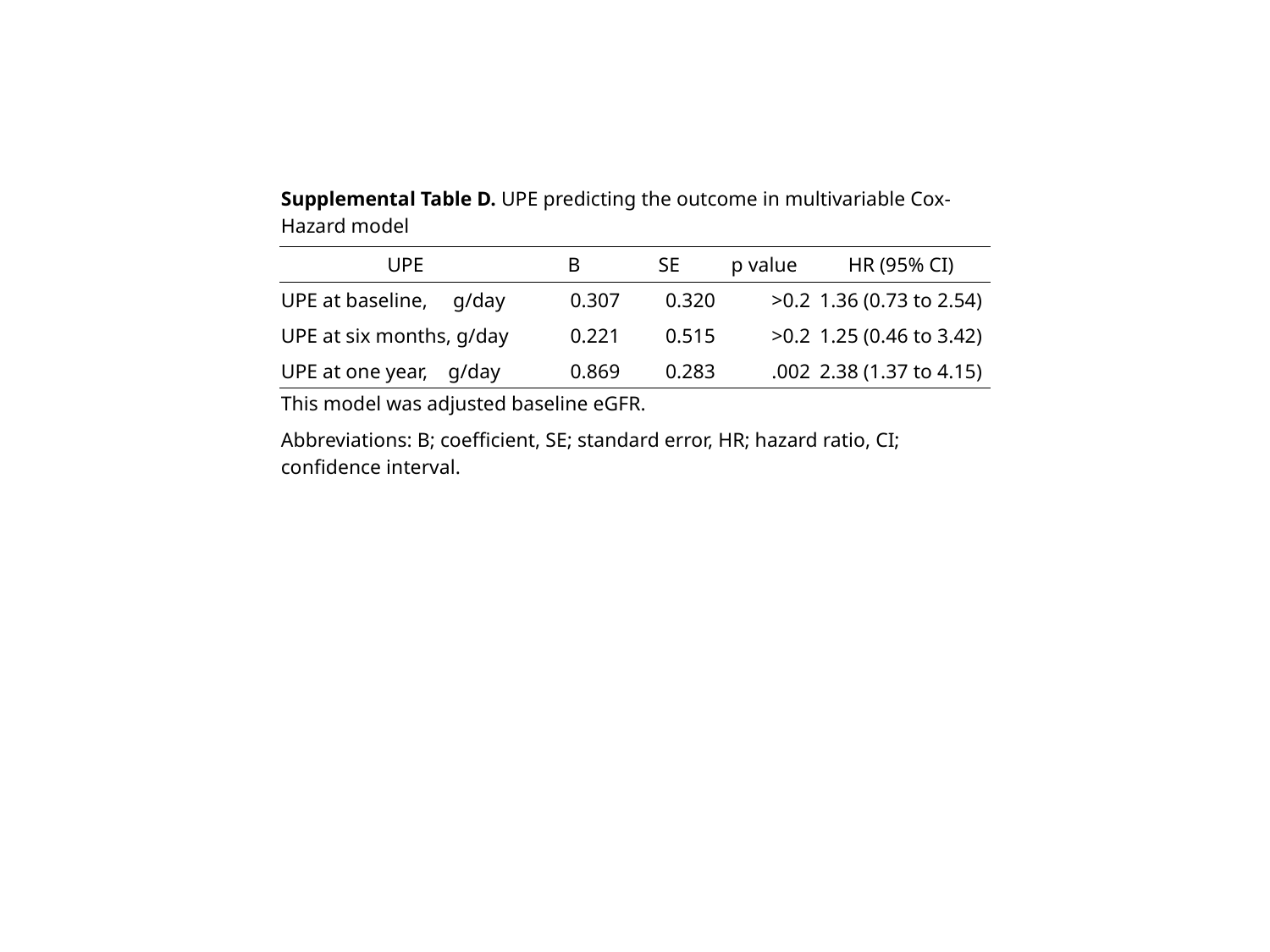

| Supplemental Table D. UPE predicting the outcome in multivariable Cox-Hazard model | | | | |
| --- | --- | --- | --- | --- |
| UPE | B | SE | p value | HR (95% CI) |
| UPE at baseline, g/day | 0.307 | 0.320 | >0.2 | 1.36 (0.73 to 2.54) |
| UPE at six months, g/day | 0.221 | 0.515 | >0.2 | 1.25 (0.46 to 3.42) |
| UPE at one year, g/day | 0.869 | 0.283 | .002 | 2.38 (1.37 to 4.15) |
| This model was adjusted baseline eGFR. | | | | |
| Abbreviations: B; coefficient, SE; standard error, HR; hazard ratio, CI; confidence interval. | | | | |

## Slide 10
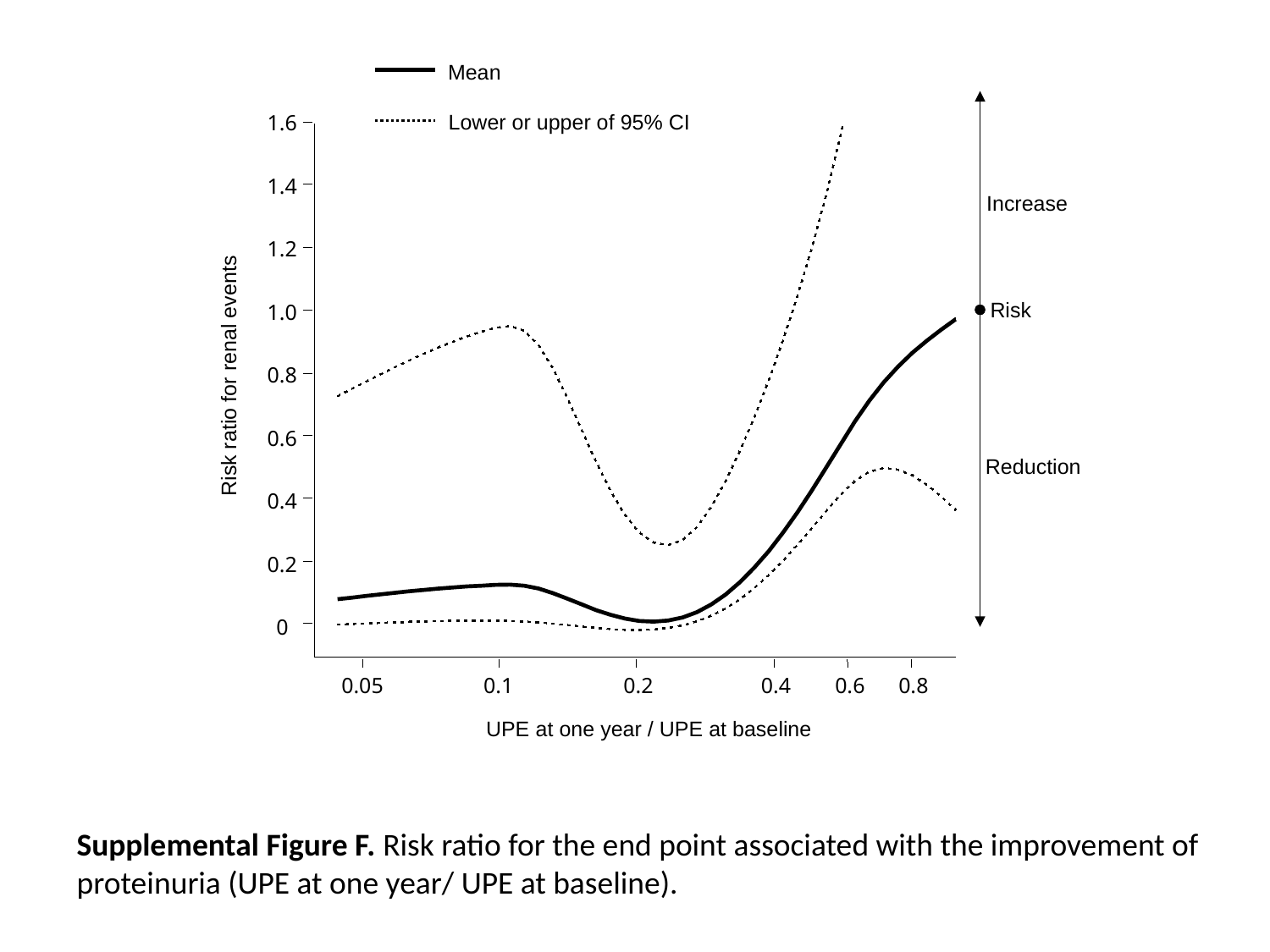

Mean
Lower or upper of 95% CI
1.6
1.4
1.2
1.0
0.8
0.6
0.4
0.2
0
Increase
Risk
Risk ratio for renal events
Reduction
0.05
0.1
0.2
0.4
0.6
0.8
UPE at one year / UPE at baseline
Supplemental Figure F. Risk ratio for the end point associated with the improvement of proteinuria (UPE at one year/ UPE at baseline).

## Slide 11
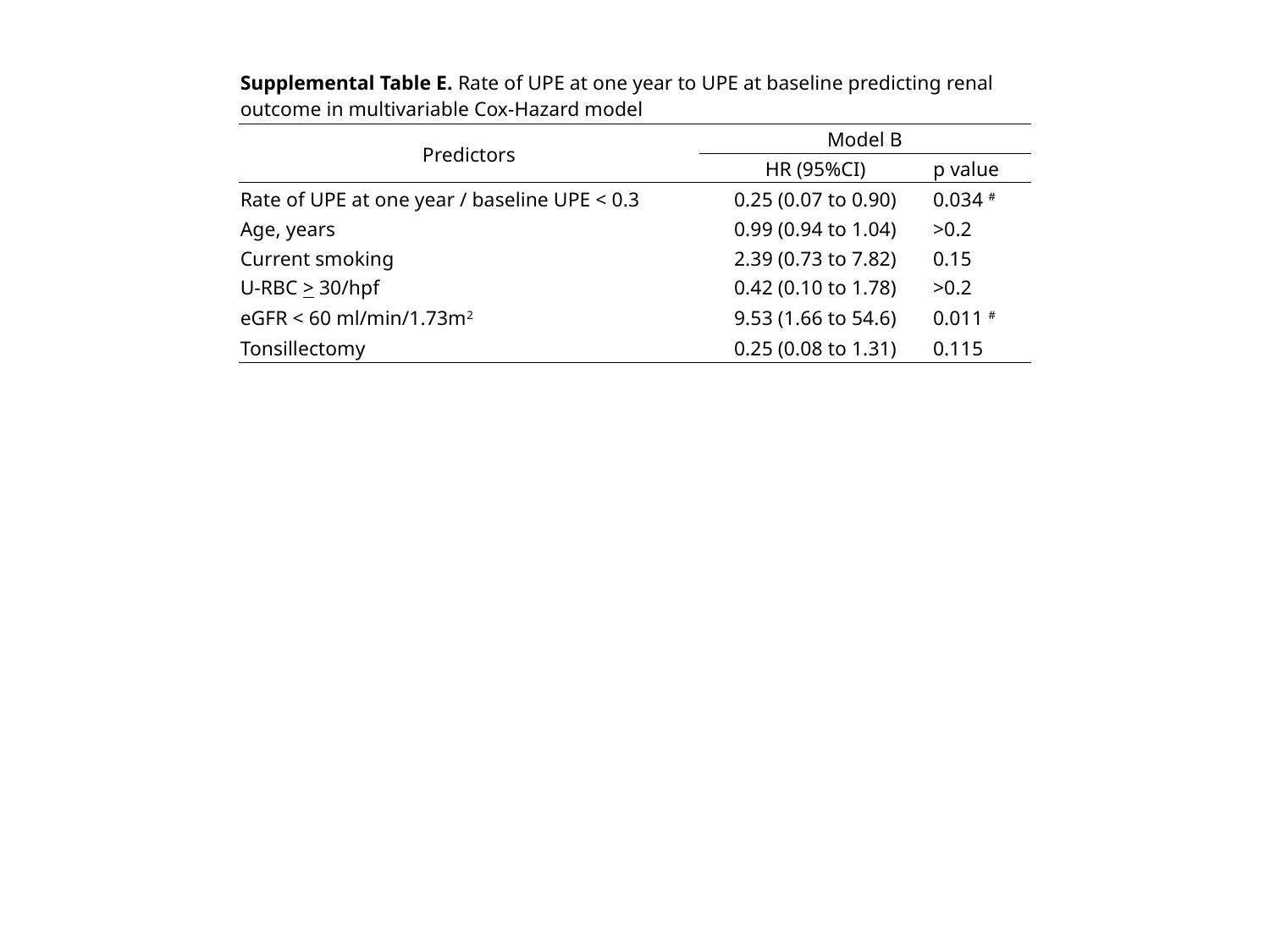

| Supplemental Table E. Rate of UPE at one year to UPE at baseline predicting renal outcome in multivariable Cox-Hazard model | | |
| --- | --- | --- |
| Predictors | Model B | |
| | HR (95%CI) | p value |
| Rate of UPE at one year / baseline UPE < 0.3 | 0.25 (0.07 to 0.90) | 0.034 # |
| Age, years | 0.99 (0.94 to 1.04) | >0.2 |
| Current smoking | 2.39 (0.73 to 7.82) | 0.15 |
| U-RBC > 30/hpf | 0.42 (0.10 to 1.78) | >0.2 |
| eGFR < 60 ml/min/1.73m2 | 9.53 (1.66 to 54.6) | 0.011 # |
| Tonsillectomy | 0.25 (0.08 to 1.31) | 0.115 |
